# Polypyrrole Nanowires with Ordered Large Mesopores: Synthesis, Characterization and Applications in Supercapacitor and Lithium/Sulfur Batteries

**DOI:** 10.3390/polym11020277

**Published:** 2019-02-07

**Authors:** Fuxing Yin, Jun Ren, Guoyan Wu, Chengwei Zhang, Yongguang Zhang

**Affiliations:** 1 School of Materials Science & Engineering and Research Institute for Energy Equipment Materials, Hebei University of Technology, Tianjin 300130, China; yinfuxing@hebut.edu.cn (F.Y.); 201631804046@hebut.edu.cn (J.R.); guoyanwu9261115@163.com (G.W.); 2 Tianjin key laboratory of materials laminating fabrication and interface control technology, Hebei University of Technology, Tianjin 300130, China

**Keywords:** mesoporous PPy nanowires, supercapacitor, Li/S battery

## Abstract

In this work, we report the preparation of polypyrrole nanowires with ordered large mesopores (OMPW) by a simple chemical polymerization method from dual templates synthesized by self-assembling silica nanospheres in porous anodic aluminum oxide (AAO) membrane channels. The obtained OMPW showed a large surface area (231.5 m^2^ g^−1^), high aspect ratio, and interconnected large mesopores (~23 nm). The OMPW was tested as a supercapacitor electrode and showed a specific capacitance of 453 F g^−1^ at 0.25 A g^−1^. A sulfur/OMPW (S/OMPW) cathode was fabricated via a simple solution method and a heat-treatment process for lithium/sulfur batteries (LSBs). The S/OMPW composite delivered a large discharge capacity reaching 1601 mAh g^−1^ at the initial cycle, retaining 1014 mAh g^−1^ at the 100th cycle at 0.1 C. The great electrochemical performances of the OMPW capacitor electrode and S/OMPW composite were attributed to the large specific surface areas and interconnected mesopores that could supply more active sites for the electrochemical reaction and facilitate mass transfer.

## 1. Introduction

Nanostructured polypyrrole (PPy) with elevated p-conjugated polymeric chains, good conductivity, and unique electrical properties has received increasing attention in various areas including energy storage and conversion, sensing, and drug delivery [1,2,3]. The morphology and architecture of PPy-based nanomaterials often affect their functionalities in particular fields. Thus, great efforts have been devoted into the expansion of PPy nanomaterials with controllable morphologies like nanowires [4], hollow nanospheres [5,6], and porous structures [7,8].

Recently, one-dimensional (1D) PPy nanostructures have attracted considerable attention in the field of electrochemical energy storage, including supercapacitors and lithium/sulfur batteries (LSBs), due to their high conductivities and novel size effects, which could increase the contact surface area between the electrode and electrolyte [9,10]. The special aspect ratio makes it easier for the structure to be fixed on the collector and more suitable for electrochemical nanodevices. However, the applications of PPy-based electrodes are so far limited due to the insufficient use of the inner layer of the electrode caused by the low surface areas. To this end, ordered mesoporous materials look promising thanks to their large Brunner−Emmet−Teller (BET) surface area and controlled mesoporous structures [7]. Thereby, PPy nanowires combining one-dimension morphologies with ordered large mesopores are promising candidates for energy-based electrode materials. In particular, mesoporous PPy nanowires could provide interconnected mesopores, enhancing active site accessibility and rapid diffusion of both reactants and products [11,12]. These features render them attractive materials for various applications.

Generally, mesoporous nanowire materials (e.g., metals [13], metal oxides [14], and carbons [15]) could conveniently be fabricated from a dual-template combining 1D porous membranes (e.g., porous anodic aluminum oxide (AAO) membranes and porous polycarbonate membranes) with a mesopore-structure-directing agent (e.g., lyotropic liquid crystal (LLC) and silica nanospheres). This dual-templating strategy provides an effective way of controlling the mesopore size and the morphology of the wires. This dual-templating technology has been employed by our group to prepare ordered mesoporous metal nanowires and carbon nanowires [13,15]. In addition, Wu et al. [14] successfully prepared ordered mesoporous Ag, Ni, and Cu_2_O nanowires incorporating LLC as the mesopore-structure-directing agent. Nevertheless, there are very few reports on the production of mesoporous PPy nanowires.

**Figure 1:**
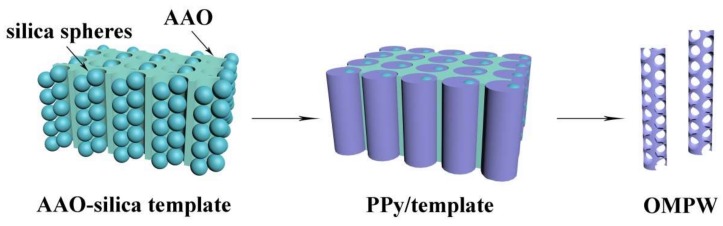
Schematic view of the synthesis route of ordered mesoporous polypyrrole nanowires (OMPW).

In this work, ordered mesoporous PPy nanowires (OMPW) are prepared by chemical polymerization using a dual-template prepared by the combination of silica nanospheres and AAO membranes. The scheme and features of this study are revealed in Figure 1. Firstly, the silica nanospheres are prepared following published methods and then deposited in the AAO template channels to produce an AAO-silica template. PPy is then filled into the AAO-silica template voids by chemical polymerization. Free standing OMPW is finally produced after etching AAO-silica with hydrofluoric acid (HF) solution. Herein, silica nanospheres are selected as assistant mesopore-forming agents because of their outstanding chemical stability and ability to control pore size in large mesoporous regions when compared to the LLC template. From the application viewpoint, PPy nanowires with large mesopores (>10 nm) are more attractive due to favorable diffusion of electrolytes when compared to platforms with small mesopores. The electrochemical properties of the obtained mesoporous PPy nanowires are tested for use in supercapacitors and as support to host sulfur in LSBs.

## 2. Materials and Methods

### 2.1. Material Synthesis

#### 2.1.1. Chemical Synthesis

TEOS (Tetraethyl orthosilicate), L-lysine, FeCl_3_·6H_2_O, and HF solution (HF, ≥40%) were from Beijing Chemical Reagents Company (Beijing, China). Sodium acetate (CH_3_COONa) and Pyrrole (C_4_H_5_N, ≥99.5%) were bought from Sinopharm Chemical Reagent Co., Ltd. (Beijing, China). Porous anodic aluminum oxide (AAO) (300 nm pore size) was from Puyuan Nano Corporation (Hefei, China). Sulfer powder was from the Sigma–Aldrich Corporation (Shanghai, China).

#### 2.1.2. Preparation of AAO-Silica Template

First, a silica nanosphere solution with uniform silica nanospheres (25 nm in diameter) was synthesized by the modified Stöber method [16]. A slice of AAO template was placed flat in a vial, then 20 mL of the above solution (5 wt%) was slowly poured into the vial and dried at 40 °C for 72 h. After complete water evaporation, AAO-silica template was obtained.

#### 2.1.3. Preparation of OMPW

A film of AAO-silica membrane was dipped in 5 mL of a solution with 0.2 mM sodium acetate and 0.2 mM pyrrole. Next, 5 mL of a solution with 0.2 mM FeCl_3_ was added at 4 °C. After polymerization at 4 °C for 12 h, the product was washed repeatedly by deionized water. The standing OMPW was obtained after etching AAO-silica template using HF solution (10 wt%, 72 h).

### 2.2. Material Characterization

The structures of OMPW were obtained by scanning electron microscopy (SEM, JEOL 6300F, JEOL, Tokyo, Japan). The porous structure and elemental analyses of OMPW and sulfer/OMPW (S/OMPW) was characterized by transmission electron microscopy (TEM, JEOL JEM-2100F, JEOL, Tokyo, Japan). The Fourier transform infrared (FTIR) spectrum was recorded with a Bruker Tensor 27 Spectrometer (Bruker, Ettlingen, Germany). X-ray diffractometer (XRD) patterns were carried out on a Rigaku D/Max-2400 (Rigaku, Tokyo, Japan). Thermogravimetric analysis (TGA, STA 409 PC Luxx, Netzsch, Selb, Germany) was performed under Ar atmosphere to determine the S content of the S/OMPW composite. Nitrogen (77 K) adsorption-desorption isotherms were conducted using a Micromeritics Tristar II 3020 analyzer (Micromeritics, Norcross, GA, USA). X-ray photoelectron spectroscopy (XPS) investigation was carried out by using a PHI model 5700 spectrometer (Physical Electronics, Chanhassen, MN, USA).

### 2.3. Electrode Preparation and Electrochemical Tests

The capacitive performance of PPy was investigated on a VersaSTAT4 potentiostat using a three-electrode system. Pt foil and a double junction Ag/AgCl (saturated KCl) electrode were employed as the counter electrode and reference electrode. The working electrodes were prepared by a slurry coating process. The active materials, carbon black and PTFE (polytetrafluoroethylene) at a mass ratio of 80:15:5, were ultrasonically dispersed in ethanol. The resulting ink was then coated on a piece of stainless steel mesh and dried at room temperature. Before testing, the foam containing the materials was compressed at 5.0 MPa. The loading of active material on each electrode was estimated to ~0.5 mg cm^−2^.

For LSBs, S/OMPW as active material was initially fabricated as follows. First, 0.1 g of the OMPW sample was dispersed in 0.4 g S/CS_2_ solution containing 0.2 g of sulfur. After continuous sonication for 2 h, the CS_2_ solution was evaporated completely. The resulting powder was then placed into Teflon-lined stainless steel autoclaves and heated at 155 °C for 12 h to further promote the uniform dispersion of sulfur powder on OMPW. Finally, S/OMPW with high dispersion of sulfur was obtained after the sample was dried at 70 °C under vacuum overnight to remove extra CS_2_.

The characterization of the electrochemical properties of S/OMPW in LSBs was performed by preparing half-cells via assembling coin-type cells (CR2032) in an Ar-filled glove box. The electrolyte consisted of 1 M lithium bis-(trifluoromethanesulfonyl)imide and 0.1 M LiNO_3_ dissolved in 1,2-dimethoxyethane and 1,3-dioxolane (1:1 *v*/*v*). Lithium metal was employed as the anode and Celgard 2400 membrane was applied as a separator. The cathode was fabricated by mixing S/OMPW composite, polyvinylidenefluoride, and acetylene black in N-methyl-2-pyrrolidinone at a mass ratio of 80:10:10 to form a uniform slurry. Then, the prepared slurry was pasted on carbon-coated Al foil followed by drying at 60 °C for 12 h. It was then cut into 15 mm diameter circular disks and pressed at 8 MPa by hydraulic press to achieve good contact between the active material and carbon coated Al foil. The active material loaded on the electrode was estimated to be ~1 mg cm^−2^, and the thickness of the electrode coating was about 0.02 mm. Thus, the tap density was calculated to be about 0.5 g cm^−3^. Cyclic voltammetry (CV) curves and electrochemical impedance spectroscopy (EIS) data were obtained by a VersaSTAT 4 electrochemical workstation. CV tests were carried out in the voltage range of 1.7–2.8 V at scan rate of 0.1 mV s^−1^. EIS data was received in the frequency range of 0.01 Hz–100 KHz with the amplitude of 5 mV.

## 3. Results and Discussion

**Figure 2:**
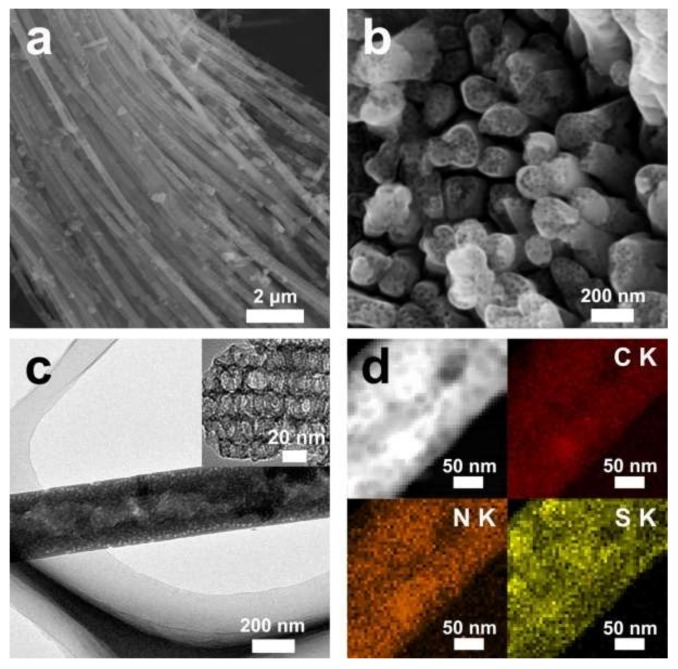
(**a**,**b**) Side-view and top-view SEM images of OMPW, (**c**) TEM image of OMPW, and (**d**) dark-field TEM image of sulfer/OMPW (S/OMPW) composite and corresponding elemental maps of carbon (red), nitrogen (orange), and sulfur (yellow).

Figure 2a shows a SEM image of OMPW, revealing aligned, densely packed nanowire arrays. The mean diameter of the nanowires was estimated to be 300 nm, which was larger than the surface pore size of the AAO template. The reason behind this may have to do with the diameter of the channels in AAO membranes, which were larger than those of the surface pores. Figure 2b exhibits the top view of OMPW, where a mesoporous structure on top of the nanowires can be easily distinguished. A TEM image of OMPW (Figure 2c) revealed a high aspect ratio and mesopores in the nanowires, suggesting complete filling of AAO channels with silica nanospheres. In an enlarged TEM view, OMPW is shown in an inset of Figure 2c and the ordered mesostructure can clearly be seen. The interconnected mesopores could favor rapid mass transport during electrochemical reactions. The large surface areas and porous structures of OMPW would make it useful as a conducting matrix for encapsulating sulfur for LSBs. Figure 2d shows a dark-field TEM image of the S/OMPW composite and the corresponding elemental mapping profiles. Uniform distributions of all three elements (C, N, and S) throughout the linear morphology were visible, demonstrating that OMPW was composed of PPy and sulfur.

**Figure 3:**
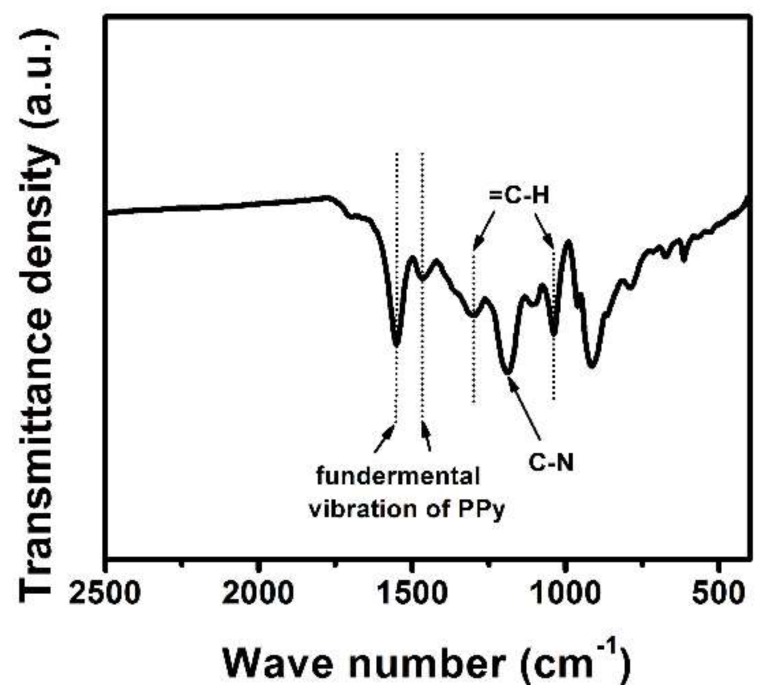
FTIR spectrum of OMPW.

Figure 3 shows the FTIR spectrum of OMPW. The FTIR spectrum demonstrated the characteristic bands of PPy [17]. From the FTIR spectrum, the characteristic bands located at 1552 and 1466 cm^−1^ corresponded to the pyrrole ring fundamental vibration, and those at 1298 and 1039 cm^−1^ aligned to the in-plane vibration of =C–H. The band at around 1196 cm^−1^ could be attributed to C–N vibration. The FTIR data indicated that PPy was successfully prepared.

**Figure 4:**
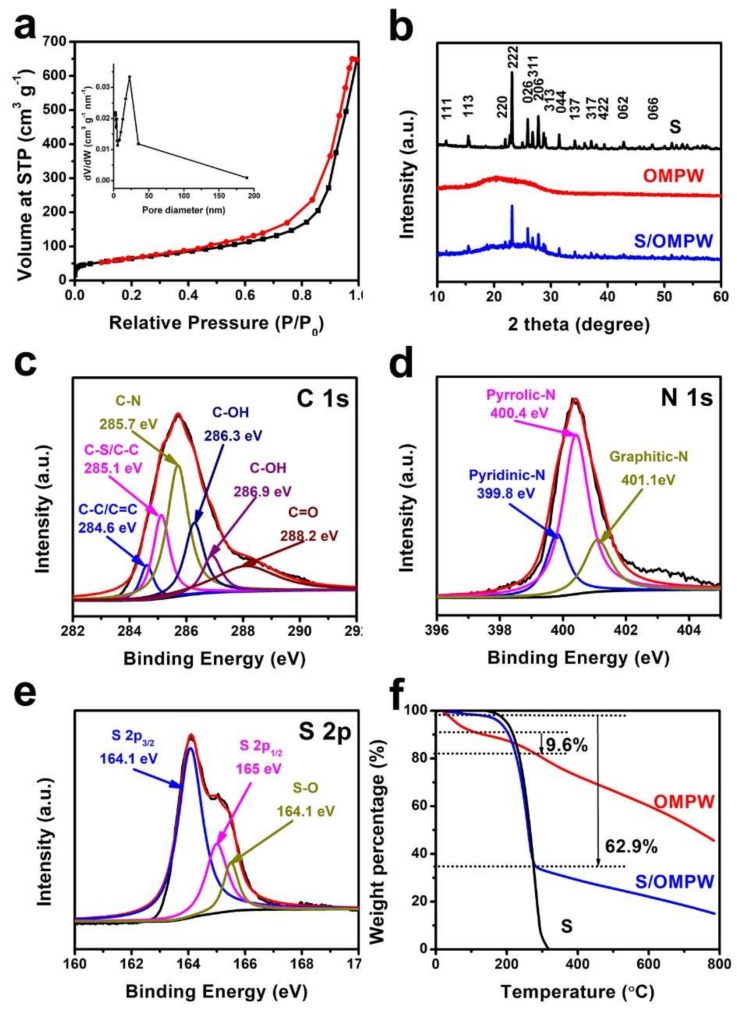
(**a**) Nitrogen adsorption-desorption isotherms and pore size distribution (PSD) curves (insert) of OMPW, (**b**) XRD profiles of S, OMPW, and S/OMPW composite, (**c**–**e**) X-ray photoelectron spectroscopy (XPS) spectra of S/OMPW composite, (**f**) TGA curves of S, OMPW, and S/OMPW composite.

Figure 4a illustrates the N_2_ adsorption-desorption isotherms of OMPW. The isotherms showed a H1 hysteresis loop, revealing that the OMPW contained mesopores. The specific BET surface area and pore volume of OMPW were calculated as 231.5 m^2^ g^−1^ and 1.03 cm^3^ g^−1^, respectively. The inset of Figure 4a shows pore size distribution (PSD) of OMPW calculated from the adsorption branch of the isotherms by the Barrett–Joyner–Halenda (BJH) method. The mesopore size obtained from the peak position of the PSD curve was 23 nm, smaller than the diameter of the original nanospheres, which resulted from the shrinkage of entire PPy frameworks caused by dehydration and solvent removal [18]. XRD was employed to investigate the crystal structures. Figure 4b depicts the XRD patterns for sulfur, OMPW, and the S/OMPW composite. For commercial sulfur, all the diffractions agreed well with the Fddd orthorhombic structure of S. By comparison, the XRD pattern of OMPW revealed a broad diffraction peak at around 2θ = 23°, which is consistent with amorphous polypyrrole. The S/OMPW composite exhibited the characteristic peaks of both sulfur and PPy, demonstrating the successful deposition of sulfur on OMPW. However, the intensity of the characteristic peak of S was obviously reduced because most crystalline S was transformed into amorphous S during heat treatment [19].

XPS was utilized to further understand the chemical composition of S/OMPW. The XPS spectrum of C 1s (Figure 4c) revealed six different fitted peaks at 284.6 eV for the C–C/C=C bond, 285.1 eV for C–S/C–C, 285.7 eV for C–N, 286.3 eV for C–OH, 286.9 eV for C–OH, and 288.2 eV for C=O. The presence of a C–O bond is mainly associated with the overoxidation of part of PPy that can break the polymer chains, producing new oxygen function on α-C [8,20]. As indicated in Figure 4d, the N 1s XPS spectrum contained three main peaks at binding energies of around 399.8, 400.4, and 401.1 eV, attributed to pyridinic–N, pyrrolic–N, and graphitic–N, respectively. Figure 4e shows two obvious peaks located at 164.1 eV (S 2p_3/2_) and 165.0 eV (S 2p_1/2_). Also, the S 2p spectrum depicts one minor peak at 165.5 eV, assigned to S–O bonds. Overall, the XPS data suggests that sulfur is covalently attached to OMPW by C–S and S–O bonds. The strong interactions between sulfur and OMPW could facilitate the cycling performances of the S/OMPW cathode in LSBs [21].

Thermal gravimetric analysis (TGA) was performed to evaluate the S loading in the S/OMPW composite. TGA curves of commercial sulfur, OMPW, and S/OMPW are gathered in Figure 4f. A rapid decomposition from 150 °C to 320 °C was observed for commercial sulfur, revealing complete evaporation of pure sulfur at temperatures below 320 °C. Below 320 °C, the TGA profiles of OMPW displayed two stages of weight loss before and after 100 °C, consistent with moisture evaporation and decomposition of OMPW. From 100 °C to 280 °C, mass loss of OMPW was recorded as 9.6 wt%, corresponding to pyrolysis of PPy. As can be observed in the TGA curve of S/OMPW, the total mass loss at the range of 100–280 °C for both OMPW and sulfur was estimated to be 62.9 wt%. Therefore, the actual content of S in the S/OMPW composite could be determined by the relationship: 62.9% = *x* + (1 − *x*) 9.6%, where *x* represents sulfur loading [22]. The actual sulfur content in the S/OMPW composite was determined to be 59 wt%.

**Figure 5:**
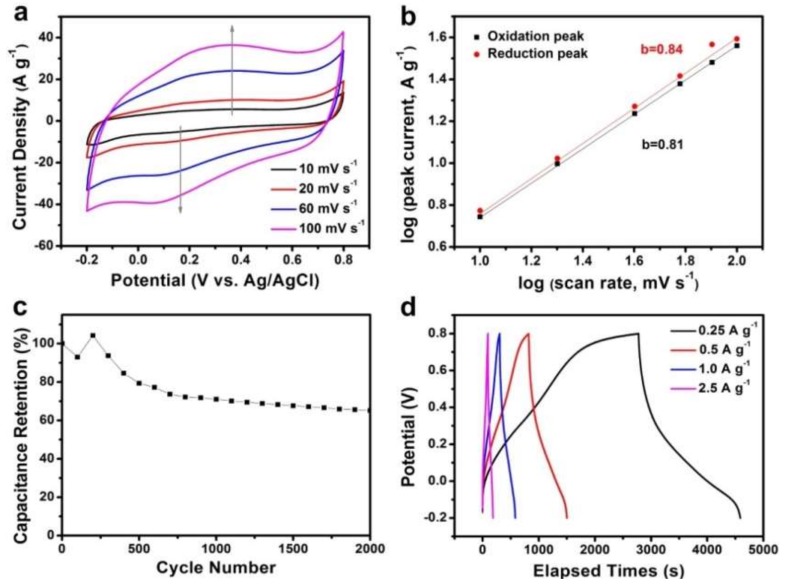
(**a**) Cyclic voltammograms (CVs) of OMPW, (**b**) plots of log (current) versus log (scan rate), (**c**) cycling performance of OMPW at a scan rate of 100 mV s^−1^, and (**d**) galvanostatic charge/discharge curves of OMPW at different current densities.

Since OMPW showed elevated specific surface area, it was used to prepare supercapacitor electrodes and tested in a three-electrode system. Cyclic voltammetry (CV) and galvanostatic charge-discharge techniques were employed to characterize the electrochemical properties of OMPW as a supercapacitor electrode. Figure 5a depicts the CVs of OMPW at different scan rates in 1.0 M H_2_SO_4_ solution in the potential range from −0.2 to 0.8 V (vs. Ag/AgCl _sat.KCl_). All CV loops of the PPy electrode presented weak redox peaks, resulting from the oxidation and reduction of PPy. The specific capacitances (*C_m_*) of the electrodes at different scan rates can be calculated from the CV curves using Equation (1) [23]:

*C_m_* = *Q*/(2*mv*Δ*V*) (1)

where *C_m_* represents the specific capacitance (F g^−1^), *Q* is the voltammetric charge integrated from CV curves, *m* is the mass of the OMPW within the electrode, *v* is the potential sweep rate (mV s^−1^), and Δ*V* is set to 1 V. The specific capacitance values of PPy reach to 435, 375, 292, and 256 F g^−1^ at 10, 20, 60, and 100 mV s^−1^, respectively. The reduction in capacitance at high scan rates is ascribed to inaccessibility of inner active sites of the electrolyte, leading the incomplete redox transitions at elevated scan rates. Therefore, the specific capacitances calculated from the CV curve at slow scan rates are considered the closest values to those of full utilization of active materials. It is possible to study electrochemical kinetics according the equation [24]:

*I* = a*v*^b^ (2)

where *i* represents the current and *v* is the sweep rate. A b-value of 0.5 suggests a total diffusion-controlled process, whereas 1 corresponds to a capacitive process. The b-value can be calculated by plotting the log (scan rate) versus log (peak current) curve (Figure 5b). The b-values were determined as 0.81 and 0.84 at voltages of respectively 0.08 V (oxidation peak) and 0.30 V (reduction peak), suggesting fast kinetics dominated by a pseudo-capacitive process [25,26].

The relatively poor cycling stability of PPy results from the continuous swelling/shrinkage of the polymer chains during electrochemical reactions, which is one of the biggest hurdles in commercial applications [3]. Here, the life cycle of OMPW was evaluated in 1 M H_2_SO_4_ electrolyte by potential (Figure 5c). During the initial 250 cycles, as the number of cycle increased, the electrode material was gradually infiltrated by the electrolyte and more and more sites were activated, resulting in an increase in capacitance [27,28,29]. The capacitive retention of OMPW after 1000 cycles was calculated as 71% of the initial value. Moreover, OMPW showed cycling stability of an estimated 8% decay from the 1000th to 2000th cycle. Because of the dissolution and/or detachment of the active materials, the specific capacitance values decreased quickly during the initial 1000 cycles. The stability of OMPW was superior compared with that of the PPy-based electrodes in previous reports, such as PPy nanowires with ~70% of their initial value remaining after 300 cycles [30], PPy/carbon aerogel composite with ~45% of the initial value after 2000 cycles [31], PPy nanowires/nanofibrous textile composite with ~61% of the initial value after 500 cycles [32], and PPy/SDPA with ~70% of the initial value after 1000 cycles [33]. The good cycling performances of OMPW was mainly due to their unique structures containing nanowires and mesopores, which would provide more stable matrices and buffer volume changes [34].

Figure 5d displays the galvanostatic charge/discharge curves of PPy. Non-ideal triangle shapes were observed and caused by the pseudo-capacitance during PPy oxidation-reduction processes. The specific capacitance (*C_sp_*) of the sample was determined from the discharge curves using Equation (3) [35]:

*C_sp_* = *I*Δ*t*/Δ*Vm* (3)

where *I* is the current (A), Δ*t* is the discharge time (s), *m* is the weight of the OMPW (g), and Δ*V* is the potential range of the discharge (V). The *C_sp_* values calculated from the curves in Figure 5d were estimated to be 453, 340, 277, and 223 F g^−1^ at current densities of 0.25, 0.5, 1.0, and 2.5 A g^−1^, respectively. These values agreed well with the CV results. Furthermore, the electrochemical performance of OMPW was higher than or comparable to that of most reported PPy-based electrodes, such as GN-PPy (165 F g^−1^ at 1 A g^−1^) [36], PPy-RGO (255.7 F g^−1^ at 0.2 A g^−1^) [37], PPy nanowires/nanofibrous textile composite (339 F g^−1^ at 0.1 A g^−1^) [32], GO-PPy (332.6 F g^−1^ at 0.25 A g^−1^) [38], GO/PPy (481 F g^−1^ at 0.2 mA cm^−2^) [39], and rGO/PPy (411 F g^−1^ at 0.2 mA cm^−2^) [40]. The outstanding performances of OMPW in supercapacitors was attributed to its high surface area and interconnected mesoporous structure that can provide more active sites and effective mass transport. Moreover, the 1D structure could facilitate ion and electron transfer.

**Figure 6:**
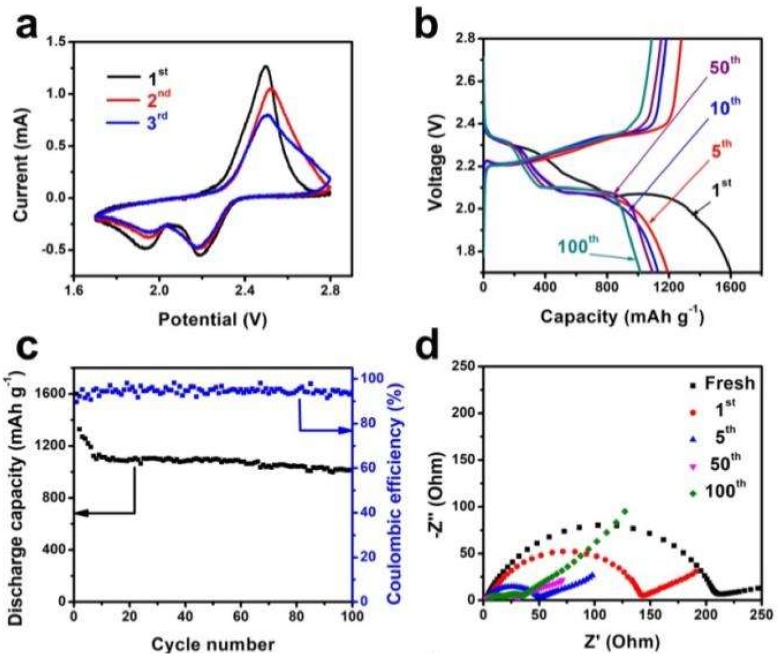

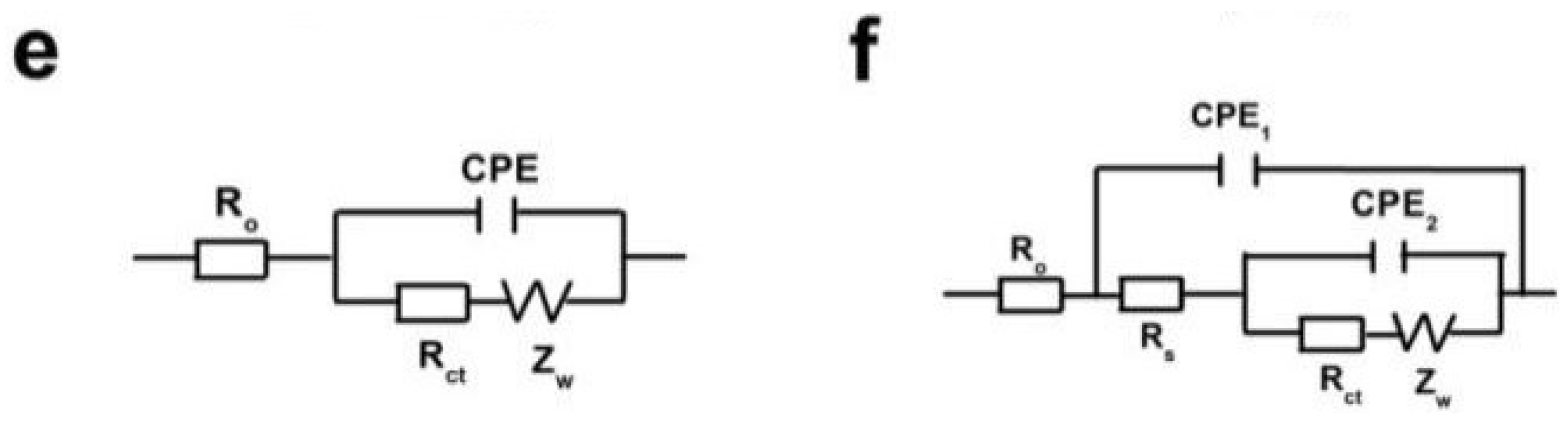
(**a**) CV curves of Li/S cells assembled with S/OMPW, (**b**,**c**) galvanostatic discharge/charge profiles and cycling test of S/OMPW at 0.1 C, (**d**) Nyquist plots of Li/S cells with S/OMPW, and (**e**) equivalent circuit of fresh Li/S cells and (**f**) cycled Li/S cells with S/OMPW.

PPy was a good conductive matrix for the sulfur cathode because PPy was proton-doped and applied as bridge to link the polymer and polysulfides anions. Thus, OMPW was applied as support to host sulfur and form the S/OMPW composite, which was then tested as a cathode material in LSBs. Figure 6a shows the initial CV curves of the Li/S cells with S/OMPW as cathodes. During the initial cathodic scan, two reduction peaks were observed at 2.2 and 1.9 V, corresponding to changes from S_8_ to long chain polysulfides (Li_2_S_x_, x > 4) and further reduction to low-order polysulfides (Li_2_S_x_, x ≤ 4), respectively. In the anodic scan, an oxidation peak appeared at 2.5 V, assigned to the reverse process [41]. The anodic peak decreased as cycling increased, indicating a decline in Lithium-storage capability of S/OMPW during initial cycling [42]. The reason behind this was the dissolution and redistribution of superficial sulfur from the OMPW surface. Figure 6b presents the discharge/charge profiles of the S/OMPW composite electrode at 0.1 C. The discharge/charge profiles were characterized by one charging and two discharging plateaus, consistent with the CV peaks (Figure 6a). The discharge/charge voltage plateaus were well-retained even after 100 cycles, suggesting the relevant recharging abilities of S/OMPW cathodes.

Figure 6c represents the cycling properties and coulombic efficiency of the S/OMPW composite electrode cycled up to 100 cycles at 0.1 C. The S/OMPW cathode delivered an initial discharge capacity of 1601 mAh g^−1^, and retained 1014 mAh g^−1^ after 100 cycles at 0.1 C. The tap density was calculated to be about 0.5 g cm^−3^. Thus, the volumetric capacity and areal capacity was calculated to be about 507 mAh cm^−3^ and 1.01 mAh cm^−2^ after 100 cycles at 0.1 C. As cycling increased, the discharge capacity decreased quickly to 1113 mAh g^−1^ after the 10th cycle. The S/OMPW cathode showed good cycling stability evaluated to have 8.9% decay from the 10th to 100th cycle. The reason for the quick capacity decay at the initial stage was linked to the dissolution of superficial sulfur from the external of OMPW. The coulombic efficiency reached over 94%, revealing efficient suppression of the shuttle effect by OMPW. The electrochemical performance of S/OMPW was outstanding compared with the other S/PPy cathodes reported in the previous literature, for instance, sulfur@polypyrrole composite (805 mA h g^−1^ remained at the 50th cycle at 0.1 C) [43], sulfur coated with polypyrrole nanolayers (>634 mAh g^−1^ remained at the 50th cycle) [44], and S/mesoporous PPy (908 mAh g^−1^ remained after 100 cycles at 0.1 C) [7]. The excellent cycling performance of the S/OMPW cathode might be attributed to the mesoporous structure of OMPW that could effectively inhibit the diffusion of polysulfides. Furthermore, the conductive PPy nanowires might enhance the charge and ion transfer within the electrode, as confirmed by the electrochemical impedance spectroscopy (EIS) measurements shown below.

Figure 6d presents the Nyquist plots of Li/S cells assembled with a S/OMPW cathode before discharge and after different cycles at 0.1 C. ZSimpWin software (Princeton Applied Research) was applied to analyze the EIS data, and equivalent circuits of fresh and cycled cells were compiled in Figure 6e,f, respectively. In this circuit, R_o_ and R_ct_ represented contact resistance and charge transfer resistance, respectively. R_s_ was the interfacial resistance corresponding to the solid electrolyte interphase (SEI) film formed on the electrode surface [8]. Z_w_ and CPE represented the Warburg diffusion and double layer capacitance, respectively. Table 1 lists the fitted impedance parameters presented in Figure 6d. The R_ct_ value decreased gradually from 197.5 Ω (fresh) to 31.9 Ω at the 5th cycle, related to the increased transportation of electrons and Li^+^ after the electrochemical activation and redistribution of non-conductive crystalline sulfur deposited on the OMPW surface during cycling [45]. The relatively stable impedance data from the 50th to 100th cycles indicated the advantages of OMPW and the combination of one-dimensional and interconnected mesoporous structures to accelerate the diffusion of Li^+^.

## 4. Conclusions

Ordered mesoporous polypyrrole nanowires (OMPW) were successfully synthesized from a dual-template (AAO-silica template) using chemical polymerization. OMPW exhibits a large specific surface area and ordered mesoporous structure, hence it was tested as supercapacitor electrodes and a sulfur host in LSBs. For supercapacitors, OMPW electrodes have fairly good specific capacitance values (453 F g^−1^ at 0.25 A g^−1^) with good cycling performance, which is attributed to its interconnected ordered mesoporous structure that can provide more active sites and effective mass transport and the fact that 1D nanostructures have more stable mechanical properties than PPy nanoparticles. For the Li/S battery, the S/OMPW cathode achieves excellent reversible capacity, high coulombic efficiency, and good cycle performance (1014 mAh g^−1^ at 0.1 C after 100 cycles). The excellent electrochemical performances can be ascribed to the unique structure of OMPW, containing ordered mesopores capable of enhancing the effective immobilization of sulfur and polysulfides. Also, the one-dimensional morphology would facilitate the electron transport. Overall, these findings indicate that OMPW is a promising electrode material for supercapacitors and sulfur support for LSBs.

## Figures and Tables

**Table 1:** Impedance parameter values extracted from the equivalent circuit.

Cycle number	R_o_ [Ω]	R_s_ [Ω]	R_ct_ [Ω]
Fresh cell	3.8	-	197.5
1st	5.2	33.5	100.3
5th	2.9	14	31.9
50th	3.2	11.4	15.7
100th	4	15.5	13.9
